# Uptake and photoinduced degradation of phthalic acid esters (PAEs) in *Ulva lactuca* highlight its potential application in environmental bioremediation

**DOI:** 10.1007/s11356-022-22142-5

**Published:** 2022-07-25

**Authors:** Dario Savoca, Riccardo Lo Coco, Raffaella Melfi, Andrea Pace

**Affiliations:** 1 Department of Biological, Chemical and Pharmaceutical Sciences and Technologies (STEBICEF), University of Palermo, Viale delle Scienze, Bd. 17, 90128 Palermo, Italy; 2 Department of Biotechnology, University of Verona, Strada Le Grazie 15, 37134 Verona, Italy

**Keywords:** Persistent organic pollutant (POP), Seaweed, Algae, Bioaccumulation, Photodegradation, UV radiation, Risk assessment, Plastic additives

## Abstract

The bioaccumulation of phthalates was studied in fragments of *Ulva lactuca* exposed for a maximum of 31 days at different concentrations of a solution of six phthalic acid esters (PAEs). The algal matrix showed rapid uptake since the first sampling, which increased over the time of the experimental period, at the end of which seaweed’s bioaccumulation potential was also evaluated. After the uptake, the algal matrix was subjected to UV irradiation in order to verify the removal of the phthalates. PAEs with higher octanol–water partition coefficients (logK_ow_) and molecular weights were preferentially uptaken by *U. lactuca* in all the exposure experiments. It was observed that both accumulation (biota-sediment accumulation factor (log_10_BSAF) ranging from 3.75 to 4.02) and photodegradation (higher than 70% removal for all phthalates in 8 h) are more efficient at lower concentration levels. These results suggest the potential use of the algal matrices for environmental bioremediation, in order to mitigate the impact of pollution from ubiquitous pollutants such as PAEs.

**Supplementary Information:**

The online version contains supplementary material available at 10.1007/s11356-022-22142-5.

## Introduction

The dispersion and bioaccumulation of persistent organic pollutants in the environment is a global concern of increasing interest, being related to the health of the ecosystems. These substances can be transported by or contained in plastic materials, from which they can gradually be released.

Today the intensive and extensive consumption of plastic (single-use or considered as such) and the resulting waste production has led this polymer to be the largest anthropogenic polluting debris in the oceans, which therefore represent its final destination (Avio et al., 2017).

As a result, despite the many beneficial applications in all areas, the development of increasingly reliable detection technologies has shown that plastic generates both physical and chemical pollution.

In fact, although plastic is not easily biodegraded, it undergoes various degradation processes (physical, chemical and biological) that lead to a very slow formation of smaller fragments (Wayman & Niemann, 2021) and to the release of additives non-covalently bonded to the polymer, such as phthalates (PAEs) (Savoca et al., 2018).

Phthalates are phthalic acid esters, which differ chemically in the substitutions of the R1 and R2 side chains (which characterise their chemical-physical properties), are slightly volatile liquids and are generally colourless and odourless (Staples et al. 1997).

Phthalates are synthetic organic chemicals typically used to confer flexibility, persistence and strength in numerous industrial applications (from films to personal care) (Wang et al. 2015). Due to these characteristics, PAEs are used both as additives in plastics (plasticisers) such as polyvinyl chloride (PVC) and as non-plasticising agents in consumer products such as perfumes or mural painting (Staples et al. 1997; Barreca et al. 2014).

Favoured by their ubiquitous diffusion (even in remote and non-industrialised areas due to oceanic and atmospheric transport (Zeng et al. 2008)), and their environmental persistence (for example 50% degradation of bis(2-ethylhexyl) phthalates (DEHP)) occurs over a year, mainly through photodegradation by the sun (Staples et al. 1997), these substances are subject to processes of bioaccumulation and biomagnification along the food chain (Cheng et al. 2021).

This contamination adversely affects the health of the organisms involved (including humans), since exposure to PAEs leads to different levels of both chronic and acute toxicity (Staples et al. 1997) mainly affecting the hormonal balance, resulting in their classifications as endocrine disrupters. PAEs are also considered risk factors for numerous multifactorial diseases (e.g. reproductive, and developmental disorders, metabolic syndromes, hepatotoxicity, oxidative stress, genetic aberrations and tumours) (Grindler et al., 2018; Rowdhwal and Chen 2018; Qian et al. 2019; Dutta et al. 2020; Li et al. 2020).

Based on these effects, in 2016, the European Chemicals Agency (ECHA) prepared a proposal to restrict the use of four phthalates: di-n-butyl phthalate (DBP), butyl benzyl phthalate (BBP), DEHP and di-isobutyl phthalate (DIBP). Announced in December 2018, the EU’s REACH restriction entered into force on 7 July 2020 and stipulated “the four substances will be limited to a concentration equal to or less than 0.1% by weight individually or in any combination” (Hilber and Gabbert, 2020).

In view of these adverse effects resulting from the exposure to phthalates and despite the regulations which have restricted or banned the use of these substances as additives, phthalate contamination will continue to cause concern due to the significance of plastic pollution.

In this context, it is necessary to assess the extent of pollution through biomonitoring studies and, above all, to adopt mitigation and compensation measures aimed at minimising the increasing dispersion of ubiquitous emerging pollutants.

However, to date, the most widely used methods of contaminant removal include a series of actions and remediation works with high economic-environmental impact and social commitment: dredging of contaminated sediment, storage operations of matrices, transfer to landfills followed by numerous treatments (Eggleton & Thomas 2004; Perelo 2010; Cheney et al. 2014).

Among the possible solutions that are not harmful to the environment and the economy, bioremediation, a set of in situ or ex situ eco-sustainable techniques, focuses on the non-invasive use of bioaccumulators or biological processes (e.g. microorganisms, fungi, plants and algae) to remove, detoxify or immobilise contaminants from the environment (Kensa, 2011).

Bioremediators such as algae are ideal candidates to carry out the processes mentioned above. In fact, the “phytoconcentration” has shown effectiveness towards a wide range of pollutants such as mercury, chlorinated solvents, PAH and PCBs (McCutcheon & Schnoor 2004; Pilon-Smits 2005).

In this context, *Ulva lactuca* possesses physico-chemical characteristics which makes this species one of the most used in biomonitoring studies both in the field and in a controlled environment (Yaich et al., 2011). In addition, this green macroalgae has a high resistance and accumulation capacity of pollutants, a wide distribution and easy reproduction.

For these reasons, this alga is an ideal model for carrying out bioremediation works from polluted environmental matrices, as already demonstrated in the literature (Cheney et al. 2014).

In addition, the algal biomass, once performed its main function, can be cleaned from accumulated compounds through green methods (photoinduced degradation, biocatalysis, use of bio-based solvents etc.), chosen according to the characteristics of the molecules to be degraded. In particular, the photodegradation process is particularly suitable for persistent and ubiquitous photosensitive organic molecules including phthalic acid esters (Barreca et al., 2013; 2014).

This process is mainly generated by the UV component of the solar radiation (UV-B and UV-A, with wavelength *λ* from 200 to 380 nm) through a series of complex chemical reactions that are specific to the structure under examination. In particular, photodegradation of phthalate esters at 254 nm involves photoinduced decarboxylation, hydroxylation, dealkylation and splitting of C-O, C–C and O-alkyl bonds (Lau et al. 2005) with a demonstrated removal efficiency of up to 90% (Wang et al. 2019). Despite the great potential application of the technique on the algal biomass, to date, there are no studies present in the literature.

The aim of this research work is to enrich the scientific knowledge related to this emerging field of interest, through the qualitative and quantitative determination in *Ulva lactuca* of six PAEs particularly widespread in environmental matrices: DMP (dimethyl-phthalate), DEP (diethyl-phthalate), DBP, BBP, DEHP, DNOP (dioctyl phthalate). *Ulva lactuca*’s ability to bioaccumulate those substances (uptake) over time has been assessed by exposure experiments conducted at different concentrations under controlled conditions for 31 days. Moreover, once verified the uptake, the efficacy of the photoinduced degradation of the chemical species accumulated in *Ulva lactuca* was evaluated. This paper represents the first uptake assessment of PAEs by *U. lactuca* in a controlled environment and the application of a sustainable technique for the removal of the same pollutants in this type of biological matrices for environmental bioremediation applications.

## Materials and methods

### Materials, equipment and software

The procedure for PAE extraction was adapted from Savoca et al. (2018). For all extraction procedures described below, only laboratory tools made of quartz, glass, ceramic or stainless steel, previously cleaned with acetonitrile (1 ×), hexane (1 ×) and acetonitrile (1 ×), were used.

Due to their different logK_ow_ in the range of 1.61 and 8.18, a standard mixture of six representative commercial phthalates in hexane (EPA Phthalate Esters Mix), containing butylbenzyl phthalate (BBP), di-n-butyl phthalate (DBP), diethyl phthalate (DEP), bis(2-ethylhexyl) phthalate (DEHP), dimethyl phthalate (DMP) and di-n-octyl phthalate (DnOP), at a concentration of 2000 mg/L (2000 ppm), was used both as a reference for calibration in the range 10–0.001 ppm before each round of analyses with linear response *R*^2^ ranged from 0.996 to 0.999, and as a surrogate added in spiked samples before extraction.

The analytical procedure (described in the supporting information) has allowed the comparison of the results obtained after the addition of standards (spike) in order to obtain the average percentage recovery (R%).

The determination of the PAEs was carried out in duplicates by analytical instrumentation Agilent Technologies 7000C GC/MS-TQ (Triple Quad) equipped with column DB-5MS (length: 30 m; diam: 0.250 mm; film: 0.25 μm; temperature limit: 350 °C).

The analyses were conducted in multiple reaction monitoring (MRM) mode with the instrumental parameters described in the supporting information and in Table S2.

The relative standard deviations (RSDs) on four replicates of quality checks were less than 5% and sample analyses were corrected accordingly.

LOD and LOQ were quantified by the IUPAC method; LOD was 0.1 ng/g for DMP and DEHP, 0.15 ng/g for DEP and DBP, 0.2 ng/g for BBP and 0.3 ng/g for DnOP; LOQ values range from 0.3 to 0.7 ppb which were determined for all phthalates.

For all determined analytes, average recoveries ranged from 71 to 164% for sediments and from 15 to 84% for *U. lactuca* (Table S1).

These recovery values were used to quantify the presence of PAEs both for the uptake evaluation, during the three samplings, and for the analysis of the removal of pollutants through photochemical processes. The latter was performed by placing quartz tubes containing the sample to be treated in a photochemical reactor Rayonet RPR-100 equipped with lamps that emit UV radiation (the main wavelength is 254 nm).

Equation 1 has been used to calculate the percentage of photodegradation in *U. lactuca* samples.

1 $$\%\left[\mathrm{PAE}\right]\;\mathrm{Removal}=100-100\times\left(\frac{\lbrack{\mathrm{PAE}\rbrack}_{\mathrm{tf}}}{{\left[\mathrm{PAE}\right]}_{\mathrm t0}}\right)$$

${\left[\mathrm{PAE}\right]}_{\mathrm{tf}}$ = PAE concentration at final time ($\mathrm{tf}$): post-irradiation

${\left[\mathrm{PAE}\right]}_{\mathrm t0}$ = PAE concentration at time zero ($\mathrm t0$): pre-irradiation

Equation 2 was used in order to determine the biota-sediment accumulation factor (BSAF) at day 31 (or 31st day), in accordance with our previous work (Savoca & Pace, 2021).

2 $$\log_{10}\mathrm{BSAF}=\log_{10}\left(\frac{{\left[\mathrm{PAE}\right]}_{\mathrm{Seaweed}}+0.001\frac{\mathrm{mg}}{\mathrm{Kg}}}{{\left[\mathrm{PAE}\right]}_{\mathrm{Sediment}}+0.001\frac{\mathrm{mg}}{\mathrm{Kg}}}\right)$$

**Fig. 1:**
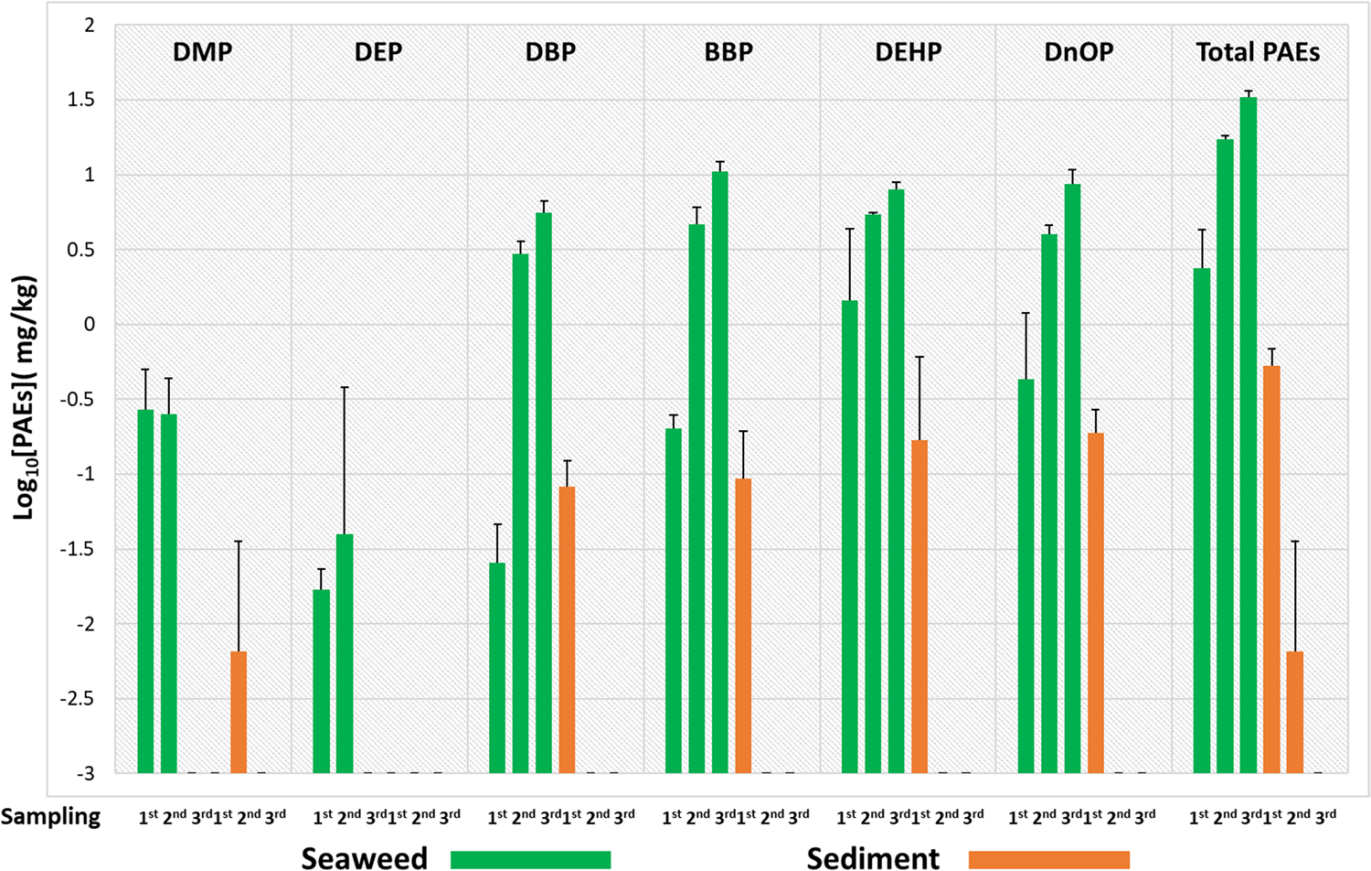
Control experiment (0 ppm): logarithmic concentration levels ± SD for each of the six phthalates and their sum (total PAEs) in *U. lactuca* samples (green box) and sediments (orange box) related to each of the microcosms analysed at three sampling points during the experimental period

For uptake graphs (Figs. 1, 3–6) and the BSAF evaluation graph, all concentration levels were transformed into logarithmic values for better graphical visualisation of trends. All the averages of the [PAEs] values have been increased by 1 ppb units in order to avoid the occurrence of indefinite values of log_10_[PAEs] for zero concentration levels. Unlike uptake graphs in which several zero values have been recorded (below the detection limit: LOD) (see log_10_[PAEs] =  − 3 in Fig. 1 and Fig. 4), for the BSAF graph, the values equal to zero were recorded only in few cases. In fact, in the control experiment for DMP and DEP in both matrices (algae and sediment), and in the same microcosms, only for sediments, these values were also recorded in DBP, BBP and DEHP, while in the 10 ppm experiment, [PAEs] were 0 for DMP and DEP only in sediments.

Although uptake graphs show values expressed in logarithmic scale, results and related discussions report unprocessed concentration values that can be derived from graphs or observed directly in Figures S1-S5 of the supplementary information.

### Experimental conditions and design

Details of the sampling campaign and pre-experiment operations are described in the supporting information.

A total of 1 g of *U. lactuca* was placed in each of the glass tubes containing 1 g of sediment contaminated at 5 different nominal concentrations of PAEs: 0 ppm (mg/kg), 5 ppm (mg/kg), 10 ppm (mg/kg), 50 ppm (mg/kg), 100 ppm (mg/kg) and 5 mL of seawater. From the standard stock solution (2000 μg/mL) through serial dilutions in hexane, four 10 mL flasks were prepared for 100 ppm, 50 ppm, 10 ppm and 5 ppm PAE solutions respectively. From these solutions, 1 mL was taken and added to each tube containing 1 g of dry sediment. The solvent was removed by a gentle flow of nitrogen (N_2_) and the content homogenised for 30'' with a vortex.

The experiment lasted a total of 31 days, with 3 monitoring sampling on days 5 (1st sampling), 12 (2nd sampling) and 31 (3rd sampling).

In order to assess the uptake pattern of seaweed from sediments during the 31 experimental days, these were analysed for each microcosm. However, *U. lactuca* samples from the 1st sampling (day 5) were used both for the uptake study and for the photoinduced degradation experiment (for the latter test only for concentrations of 0, 10 and 100 ppm PAEs). While the uptake experiment was conducted at least in duplicates, for photoinduced degradation tests (in which uptake was also evaluated), considering the 3 times of exposure to radiation (0.5 h, 1.5 h, 8 h), six more tubes were predisposed (#1–6 per control, #17–22 per experiment at 10 ppm and #33–38 per 100 ppm). In all cases, all concentration values reported in the “Results and discussion” refer to the average values. The experimental setup consisting of 42 tubes is shown in Table S3 of the supporting information.

### Samples preparation and PAE extraction

The two matrices (sediment and alga) contained in each test tube of the experimental setup were extracted with the same analytical procedure, in order to specifically assess the accumulation, the temporal distribution and the levels of PAE concentration after treatment with UV irradiation for the removal of the pollutants.

Briefly, the two components of each system (algae fragments and sediments) were transferred into clean test tubes and underwent a freeze-drying process (ScanVac CoolSafe dryers) to eliminate the water content and to avoid analytical interference due to the presence of salts in aqueous solution; for this reason, all the concentration values for the extractions are expressed in dry weight.

In particular, *U. lactuca*’s fragments were weighed with an analytical balance to assess the weight of the dehydrated biomass (the average loss of water content of the samples was 87.4% ± 0.2).

Once freeze-dried, the PAEs of the individual matrices were extracted at room temperature with 2 mL of acetonitrile (AcN), in an ultrasonic bath for 20 min and then centrifuged for 10 min at 4000 rpm min^−1^ (LaboGene ScanSpeed 416); an aliquot of supernatant was transferred to transparent 1.5 mL glass vials for GC/MS analysis. In the specific case of 50 and 100 ppm exposure samples, at the end of the extraction process and before chromatographic analysis, the supernatant aliquot obtained was diluted 1:10 with AcN.

The lyophilised *U. lactuca* fragments contained in each test tube relative to the 1st sampling and subjected to PAE contamination at 0 (control), 10 and 100 ppm were divided into 2 parts to be used both for the uptake evaluation (time zero) and for the PAE photoinduced degradation tests by UV irradiation in quartz tubes.

## Results and discussion

This study provides the first contamination data on the uptake of PAEs under controlled conditions in *U. lactuca*, and its subsequent treatment of photodegradation.

### Chemical analyses of uptake of PAEs in *U. lactuca* thalli 

For each exposure level and sampling day, seaweeds and sediments were analysed in order to examine the partitions of phthalates. In addition, the different values of the octanol–water partition constants (logK_ow_) (Ellington 1999) intrinsic to each phthalic ester were correlated with the seaweed uptake results obtained. The collected data showed that phthalates with higher logK_ow_ values were present at higher concentrations (see Fig. S1-S5 in the supporting information).

The chemical analysis of phthalate acid esters concentration ([PAEs]) in seaweed exposed (or not) to different concentration levels of spiked sediments showed high bioaccumulation levels of the pollutants, with higher values in experiments with lower doses of PAEs.

In the control experiment (Fig. 1), the first sampling showed that the sediments were contaminated by PAEs, in particular, the sum of phthalates (total PAEs) was 0.53 ppm and the highest concentrations found were for BBP, DEHP and DnOP with ranges from 0.09 to 0.19 ppm.

Surprisingly, there was a decrease of 95% in the total PAEs in the sediment between the first and the third sampling, and a consequent increase in *U. lactuca* of approximately 14 times. The highest concentrations recorded during the second sampling concerned DBP, BBP, DEHP and DNOP ranging from 2.96 ppm for DBP to 5.45 ppm for DEHP; while during the third sampling, these four phthalates were the only ones to be detected, ranging from 5.6 ppm for DBP to 10.56 ppm for BBP.

**Fig. 2:**
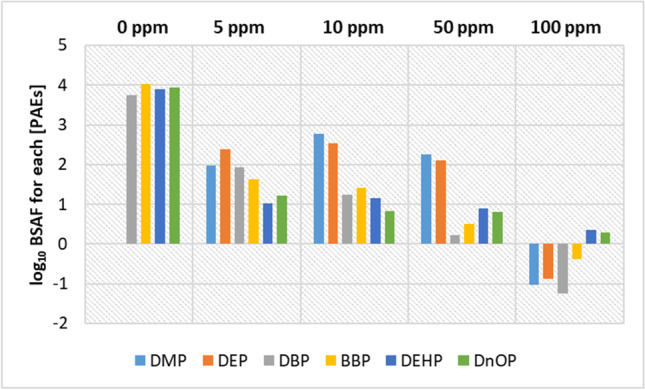
Graph showing the log_10_BSAF values calculated at the end of experiments (31 days) for each PAE and exposure concentrations (at the top of the *x*-axis: 0 ppm, 5 ppm, 10 ppm, 50 ppm and 100 ppm)

**Fig. 3:**
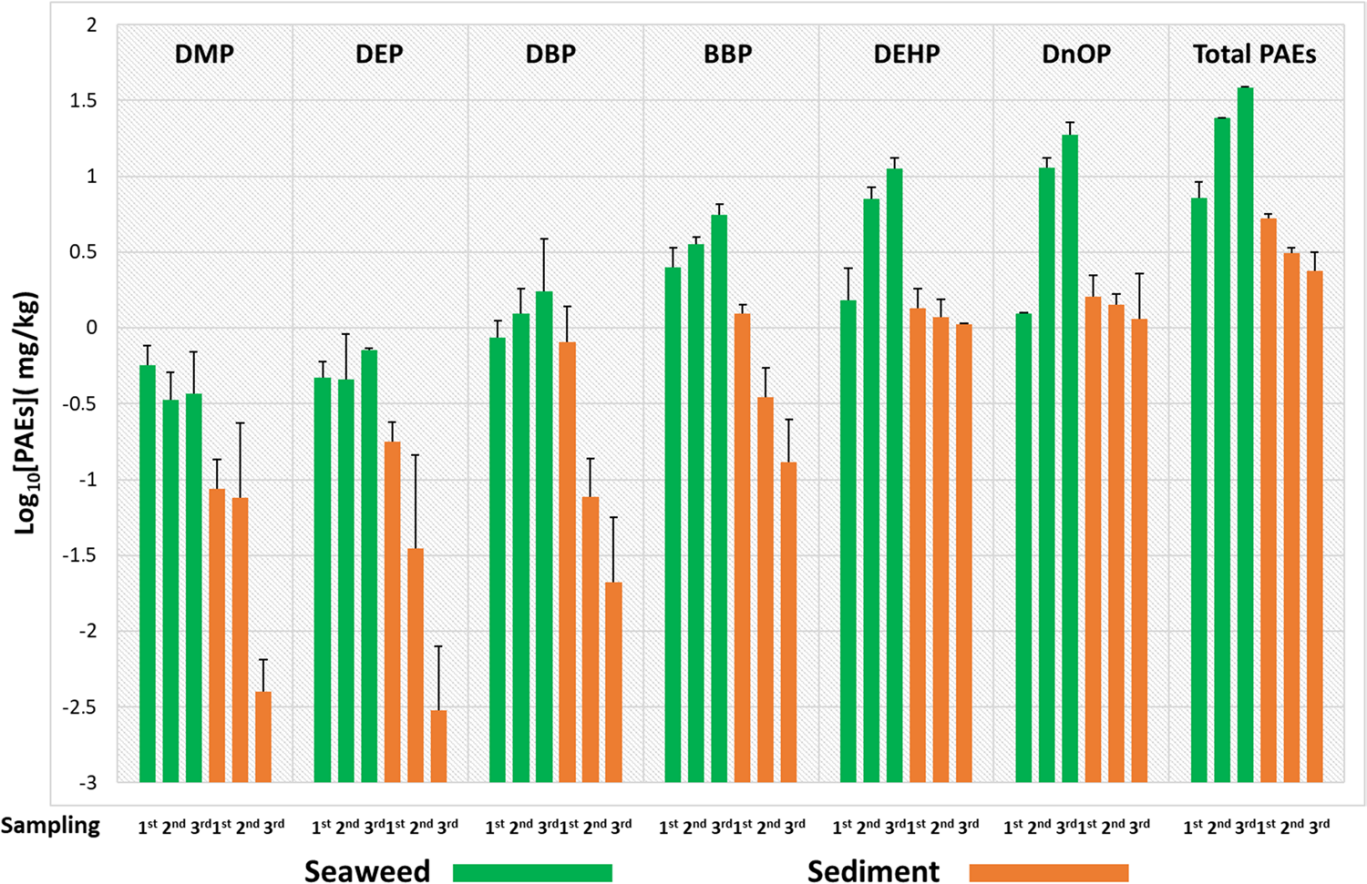
5 ppm experiment: logarithmic concentration levels ± SD measured for each of the six phthalates and for their total sum (total PAEs) in *U. lactuca* samples (green box) and sediments (orange box) related to each of the microcosms analysed at three sampling points during the experimental period

**Fig. 4:**
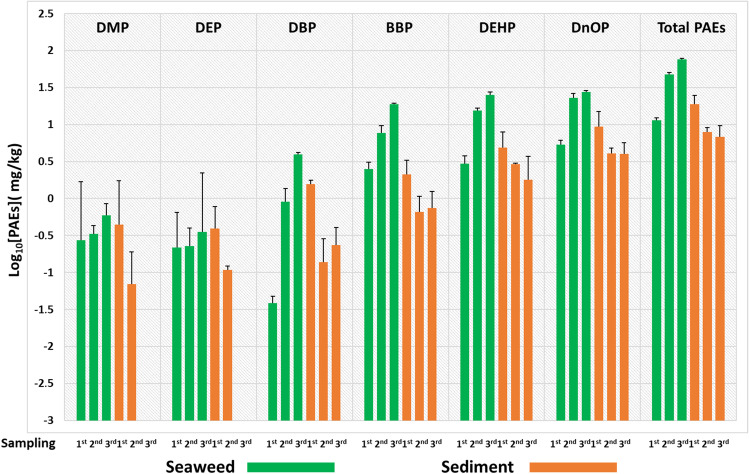
10 ppm experiment: logarithmic concentration levels ± SD measured for each of the six phthalates and for their total sum (total PAEs) in *U. lactuca* samples (green box) and sediments (orange box) related to each of the microcosms analysed at three sampling points during the experimental period

**Fig. 5:**
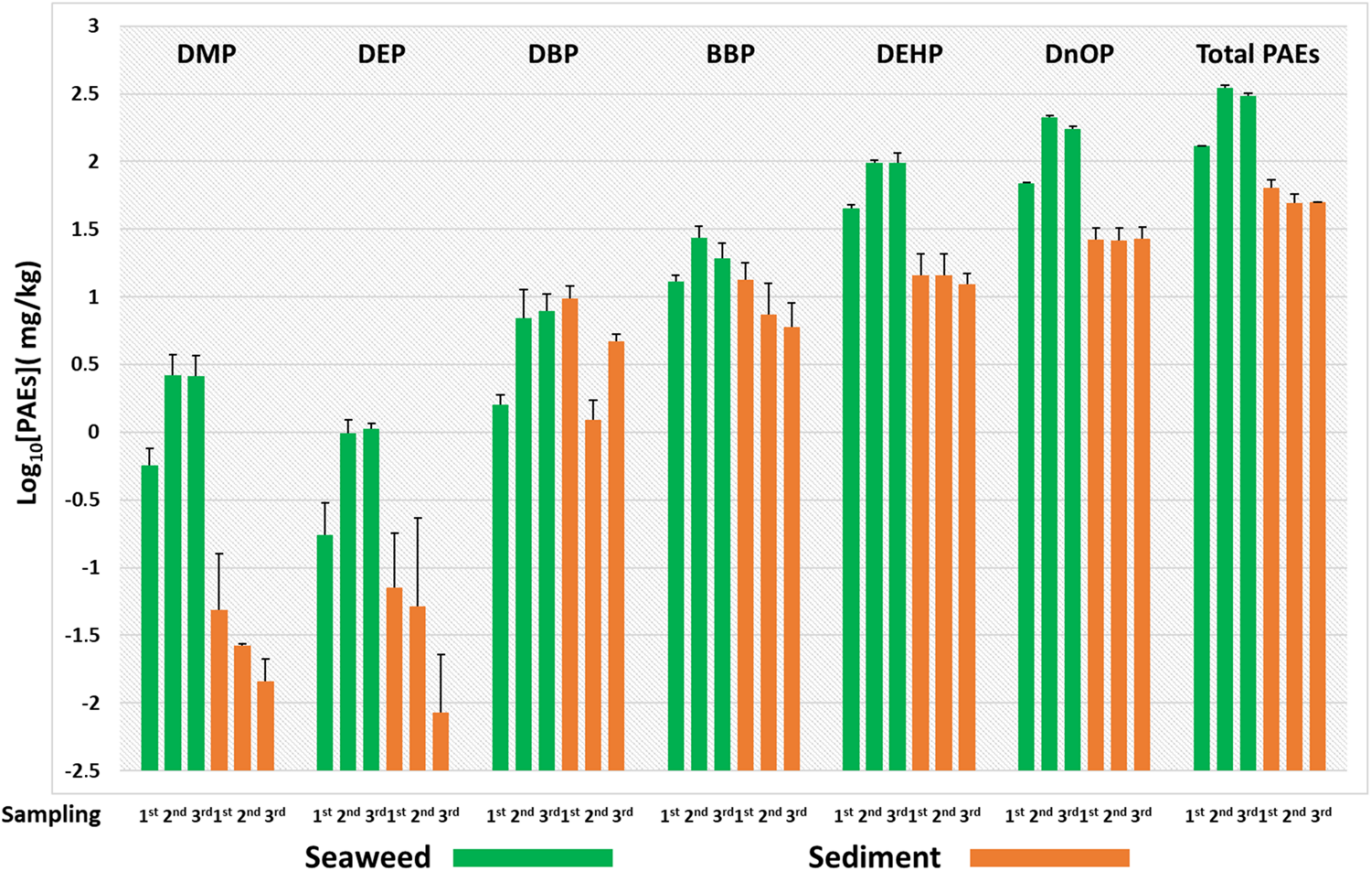
50 ppm experiment: logarithmic concentration levels ± SD measured for each of the six phthalates and for their total sum (total PAEs) in *U. lactuca* samples (green box) and sediments (orange box) related to each of the microcosms analysed at three sampling points during the experimental period

Furthermore, although the initial concentrations in the control microcosm were lower than in all other exposure experiments (Figs. 1–5), the highest values of log_10_BSAF (range: 3.75 to 4.02) were recorded at the end of the experimental period, with the exception of DMP and DEP (Fig. 2), which were not detected in the sediments probably due to very low initial concentrations (while in seaweed were 0.27 ppm for DMP and 0.02 for DEP) and/or biodegradation processes (Net et al. 2015).

Such elaboration has shown a high degree of PAE bioaccumulation in the algal matrix. In particular, on the 31st day, the highest log_10_BSAF values of the most lipophilic PAEs (DBP, BBP, DEHP, DNOP) were recorded in the control experiment, while in the other microcosms, the values progressively decreased as the exposure concentration increased.

In this context, interestingly, high log_10_BSAF values (range 0.83–2.78) were also recorded in 5 and 10 ppm experiments; however, the highest values were related to DEP and DMP.

However, deepening the analysis of all those matrices exposed to the PAE standard mix, the exposure to 5 ppm was the lowest to which the microcosms were subjected and the one where a high capacity of *U. lactuca* to bio-accumulate and concentrate the added esters was observed (Fig. 3).

The results obtained show an exponential trend in the time span ranging from the 1st to the 3rd sampling point, with a decrease of 53% of total PAEs in sediments.

Although total PAE accumulation of 7.15 ppm was detected in *U. lactuca* samples in the first sampling (the maximum recorded value for BBP was 2.5 ppm), the highest uptake rate was recorded between the second and third sampling, showing a total PAE concentration of 24.14 ppm and 38.54 ppm respectively. In particular, in these last two samplings, the highest levels of PAEs were recorded for DEHP (7.12 ppm in the 2nd sampling and 11.29 ppm in the 3rd sampling) and for DNOP (11.45 ppm in the 2nd sampling and 18.84 ppm in the 3rd sampling).

Similarly, the experiment of exposure to 10 ppm (Fig. 4), from the 1st to the 3rd day of sampling, showed a decrease of 64% in the concentration of total PAEs in sediments.

In this experiment, the first sampling showed higher concentration levels in the sediments than in the algae (total PAEs in seaweed were 11.33 ppm vs 18.74 ppm in sediments) with the exception of BBP, while in the second sampling, there was a significant increase of [PAEs] in *U. lactuca* and a consequent decrease in the sediments. In fact, during the 2nd sampling, total PAEs in seaweed were 47.87 ppm while in sediments 7.96 ppm, while during the 3rd sampling, the recorded values were 76.2 ppm in seaweeds and 6.8 ppm in sediments. In both matrices, analyses showed that the largest contribution was from BBP, DEHP and DNOP.

**Fig. 6:**
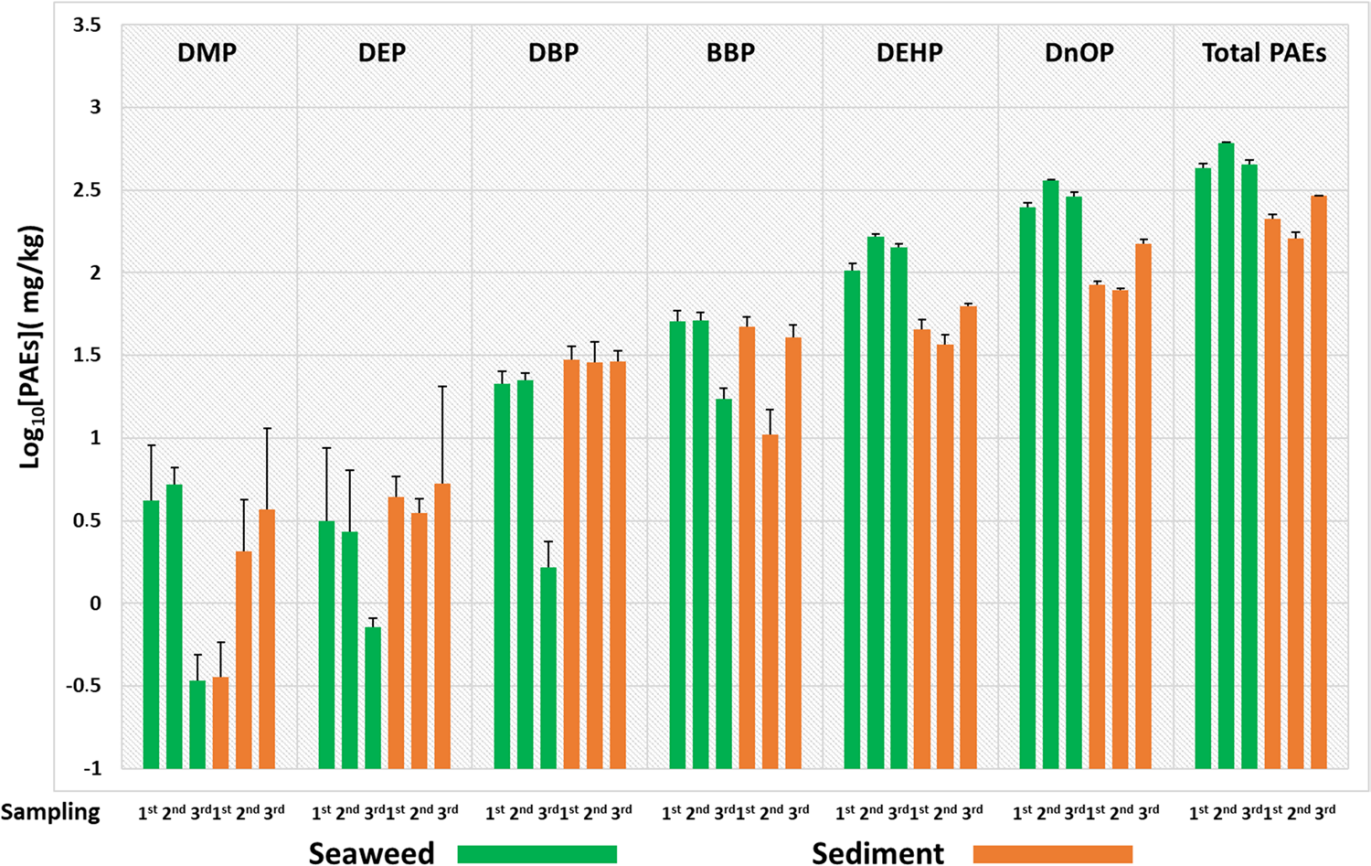
100 ppm experiment: logarithmic concentration levels ± SD measured for each of the six phthalates and for their total sum (total PAEs) in *U. lactuca* samples (green box) and sediments (orange box) related to each of the microcosms analysed at three sampling points during the experimental period

In microcosms where sediments were prepared at concentrations of 50 (Fig. 5) and 100 ppm (Fig. 6), a high uptake rate by *U. lactuca* was recorded between the first (total PAEs were 129.34 ppm for the 50 ppm experiment and 430.58 ppm for the 100 ppm experiment) and the second day of sampling (total PAEs were 346.95 ppm for the experiment at 50 ppm and 608.37 ppm for that at 100 ppm). Instead, on the third sampling, the trend of increasing concentration levels of all six PAEs in algae was not observed.

In fact, on the first day of sampling, high values were recorded for DEHP and DNOP: 45.21 and 68.79 ppm respectively for the 50 ppm experiment (Fig. 5), while 102.78 and 248.79 ppm for the 100 ppm experiment (Fig. 6).

Similarly, for the second day of sampling, the highest levels were found for DEHP and DNOP with values of 97.66 and 211.3 ppm respectively for the experiment at 50 ppm (Fig. 5), while for the 100 ppm experiment were 165.22 and 361.55 ppm (Fig. 6).

However, on the following sampling day (third), DEHP and DNOP concentration values decreased for both microcosms (more for the experiment at 100 ppm than for the experiment at 50 ppm).

Indeed, during the whole experimental period, the sediments did not show a high decontamination rate of total PAEs which was observed between the first and second day of collection (23.4% for the 50 ppm experiment and 24.5% for the 100 ppm experiment). In fact, in this matrix, between the second and third day of sampling, the total concentration levels of phthalates remained similar for the experiment at 50 ppm (from 49.1 to 49.67 ppm), while they increased in the experiment at 100 ppm (from 159.97 to 290.628 ppm) probably due to the release of contaminants from the algae matrix to the sediments. Noteworthy, in both microcosms, all algal matrices died between the 2nd and the 3rd day of sampling as indicated by a strong odour characteristic of their decomposition (Nedergaard et al. 2002).

The dangerousness of phthalates derives from their ability to interact with cell membranes, which is justified by their affinity to organic portions, represented by the different values of the octanol–water partition coefficient (logK_ow_) which provides an estimate of the hydrophobicity measurement of a given chemical species. PAEs with higher values of logK_ow_ are more concentrated in organic portions, resulting in negative effects on the health of the organism involved as observed for other vegetal organisms (Adams et al. 1995; Staples et al. 1997; Jonsson & Baun 2003; Net et al. 2015; Gu et al. 2017).

In this study, of all the phthalic esters present in the microcosms, DEHP and DNOP had the highest concentration levels as similarly observed by Chan et al. (2004) for algae that showed more biosorption of DEHP, phthalate most used in Europe in the 1990s (Peijnenburg, 2008).

On the other hand, both in the natural environment and under controlled conditions, higher values of bioconcentration factor (BCF) were recorded for phthalates with higher logK_ow_ values and molecular weight such as DEHP and DNOP on the contrary to DEP and DMP that show low concentration levels (Net et al. 2015) as observed in this study.

Moreover, the low presence of DMP and DEP could be due to their relatively high water solubility (4200 and 1100 mg/L respectively) and therefore to their potential transfer from sediments to the aqueous phase, unlike DBP and BBP which are moderately adsorbed (water solubility 11.2 and 2.7 mg/L respectively), especially the poorly soluble DEHP (water solubility 0.003 mg/L) and DNOP (water solubility 0.0005 mg/L) (Staples et al. 1997).

In addition, DMP and DEP can be more easily biodegraded over time (Net et al 2015) and/or photodegraded (Barreca et al., 2014). For example, the DEP (detected in aquatic biota at modest levels) is not usually biomagnified because organisms rapidly degrade it (for example in fish where it shows two half-life days) (Net et al. 2015).

Finally, considering the significant PAE seaweed uptake recorded for all exposure levels, the first sampling point was chosen to run parallel photodegradation tests for control (0 ppm), 10 and 100 ppm experiments.

### Photodegradation of PAEs in *U. lactuca *thalli

Although few studies are available on the photodegradation of phthalic esters without the use of catalysts (Barreca et al. 2014; Lau et al. 2005; Wang et al. 2019), it has been shown that PAEs can undergo degradation by direct photolysis (absorption of UV radiation) or indirectly by reaction to activated species (singlet oxygen or hydroxyl radicals) produced by the interaction between UV radiation and natural substances present in water (Staples et al. 1997). Our study focused its attention on direct photodegradation in a dried algal matrix.

**Fig. 7:**
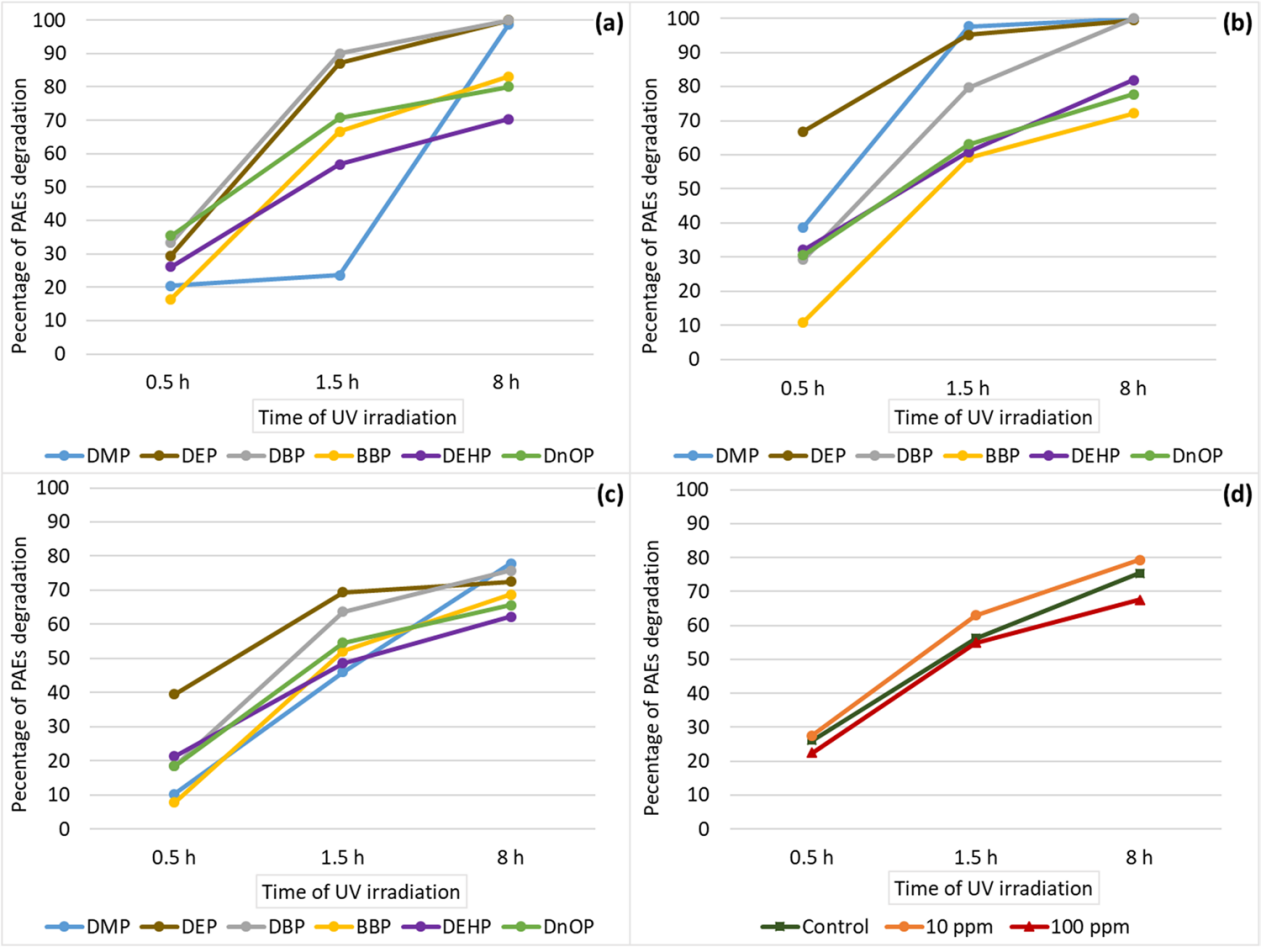
Averages of photodegradation percentages at three different irradiation times: 0.5, 1.5 and 8 h (*x*-axis) of each PAEs accumulated in algae after their exposure to three experimental concentration levels: 0 ppm (**a**); 10 ppm (**b**); 100 ppm (**c**); for these three levels, the percentage of photodegradation of the total of six PAEs is also shown (**d**)

In the present study, the algae not exposed (control) or exposed to concentrations of 10 and 100 ppm, which were taken on the 1st sampling (day 5) for uptake evaluation, are also used for photodegradation tests of phthalates at irradiation time of 0.5, 1.5 and 8 h (Fig. 7).

In seaweeds not exposed to PAEs (although contaminated, as noted in the previous paragraph) at 0.5 h, the percentage of PAE removal was between 16.4% (for BBP) and 35.4% (for DNOP) (Fig. 7a), while the total removal of the six PAEs was 26.1% (Fig. 7d). In the same experiment, after 1.5 h of UV radiation emission, the percentage of removal relative to the total of the six PAEs increased to 56.1% (Fig. 7d), where the minimum percentage of degradation value was recorded for DMP (23.6%) and the maximum for DBP (90%) (Fig. 7a). Similarly, after 8 h of irradiation, for all phthalates, an increase in percentage removal values was observed, from 70% of DEHP to 100% of DEP and DBP (Fig. 7a), with a photodegradation average for all phthalates of 88.7% and a degradation percentage of total PAEs of 75.3% (Fig. 7d).

Similarly, for the algae of the 10 ppm experiment, an increase in degradation was observed after 0.5 h. In fact, at this time of irradiation, photodegradation was recorded from 11% (for BBP) to 66.9% (for DEP) with the removal of total PAEs of 27.6%. At 1.5 h instead, the latter percentage increased to 63% (minimum 59.2% for BBP and maximum 97.7% for DMP) to further increase at 8 h of irradiation to 79.4% (minimum 72.2% for BBP and maximum 100% for DBP) with an average percentage removal for the six PAEs of 88.5%.

Algal matrices exposed to 100 ppm showed lower percentages of PAE removal after photodegradation. In fact, at 0.5 h, the removal of total PAEs was 22.5% (minimum 10.2% for DMP, maximum 39.5 for DEP) while at 1.5 h, it was 54.8% (minimum 46% of the DMP, maximum 69.4% of the DEP). Finally, at 8 h of UV exposure, total PAE removal was 67.6% (minimum 65.6 for DNOP maximum 77.9% for DMP) with an average removal of 70.5% for the six PAEs.

These results are in line with those obtained by Barreca et al. (2014) who observed a significant removal (68%) of the total PAEs on mural painting achieved by an irradiation period of 8 h.

In our study, it was observed that photodegradation is more efficient in control algal matrices and in those exposed to 10 ppm concentration. In general, it was observed that a lower concentration of PAEs in *U. lactuca* corresponded to higher efficiency of photodegradation. Probably the high concentrations of PAEs determine the inhibition of the photoinduced degradation process due to the number of molecules adsorbed and present in the treated matrix that shields the UV irradiation.

In fact, both in the control algae and in those 10 ppm exposure experiment, after 8 h of irradiation, DMP and DEP (together with DBP) are removed almost completely from the algal matrix (range 98.78–100%) while in the exposure experiment at 100 ppm, although high, the removal for these 3 PAEs was lower (range 72.47–77.9%). In the same way for BBP, DEHP and DNOP at the same time of irradiation, the percentage of removal was higher for the control and 10 ppm experiment (range 70.12–83.06%) compared to 100 ppm (range 62.25–68.81%).

In the light of these findings, and considering that on average phthalates tend to bioconcentrate more in vegetal organisms than in animals (Staples et al. 1997), the potentially effective use of *U. lactuca* is highlighted in this paper, even in heavily polluted environments (e.g. 10 ppm microcosm). In this way, it would be possible to adopt bioremediation measures for the removal of PAEs and other pollutants from the environment as seen for PCB in Cheney et al. (2014). The use of green techniques of photoinduced degradation could also eliminate the pollutants from the algal matrices where they accumulated.

## Conclusion

Phthalic esters represent a growing and current threat to human health and ecosystems due to their ability to bioaccumulate in the tissues of organisms, highlighting the need to monitor the distribution of these substances along the trophic chains and to find green and innovative processes for their removal from the environmental matrices.

Although not in the Stockholm list, phthalates share many of the characteristics of the chemicals the list contains and can be considered persistent organic pollutants.

In this sense, the ingestion of contaminated organisms that are at the base of the food chain (for example algae) can lead to the contamination of the entire trophic network.

In this context, the uptake results show higher concentration levels for chemical species with a higher logK_ow_ value (BBP, DEHP, DNOP) and are therefore potentially more biomagnificable.

This work shows how the higher uptake capacity of *U. lactuca*, as well as its higher photodegradation rates, is found in the experiment conducted at lower concentrations (closer to the environmental ones). Considering that *U. lactuca* showed high adsorption in a short time of exposure (5 days) and that a high percentage of PAEs were effectively photodegraded in this matrix, these results show potential, promising and rapid applications of bioremediations. Despite preliminary tests, this study lays the foundations for further research in the development and optimization of bioremediation system processes for field application such as photodegradation under different conditions (e.g. in aqueous solution) and investigations useful for the identification of the transformation products of these post-irradiation pollutants.

These promising results could lead to the application of these processes to restore ecosystems exposed to PAEs and all those pollutants easily adsorbed and/or photosensitive. This approach could also form the basis of a virtuous cycle of algal biomass reuse, where the key to solving the problem is scientific research, the development of green technologies and nature itself.

## Supplementary Information

Below is the link to the electronic supplementary material.

Supplementary file1 (DOCX 1141 KB)
