# Supplementary material for: Uptake and photoinduced degradation of phthalic acid esters (PAEs) in Ulva lactuca highlight its potential application in environmental bioremediation
Source: Environ Sci Pollut Res Int. 2022 Jul 25;29(60):90887–97. doi: 10.1007/s11356-022-22142-5 (PMC9722868; doi:10.1007/s11356-022-22142-5)
Supplement: Supplementary file 1 — Supplementary file1 (DOCX 1141 KB) [file 11356_2022_22142_MOESM1_ESM.docx]

**Supporting Information**

**Uptake and photoinduced degradation of phthalic esters (PAEs) in *Ulva lactuca* highlight its potential application in environmental bioremediation**

*Dario Savoca^1^, Riccardo Lo Coco^2^,* *Raffaella Melfi^1^, and Andrea Pace^1^*

^1^Department of Biological, Chemical and Pharmaceutical Sciences and Technologies (STEBICEF), University of Palermo, Viale delle Scienze, Bd. 17, 90128 Palermo, Italy;

^2^Department of Biotechnology, University of Verona, Strada Le Grazie 15, 37134 Verona, Italy

*Corresponding author: [dario.savoca@unipa.it](mailto:dario.savoca@unipa.it); Tel: +393293613086

**Total SI-Figures: 5**

**Total SI-Tables: 3**

**Total SI-Pages: 11**

**Analytical procedure for calculation of recovery percentage and concentrations**

The spiked samples of each type of matrix (seaweed, sediments) underwent the same extraction procedure as the corresponding samples of the uptake/photodegradation experiments. The recovery percentages (R %) were checked for each test batch by adding different amounts of analytical standards to blank samples prior to the extraction procedure; In particular, triple recovery tests were carried out for algal matrices and sediments, adding the standard to the samples in order to obtain final nominal concentrations of 5, 10, 50, 100 ppm.

Recovery percentage has been calculated for each phthalic ester, examined, and fortified complex matrix type (*U. lactuca*, sediments), according to equation S1.

R% = 100 * (D_spiked_ - D_unspiked_) / C_spike_  (eq.S1)

D_spiked_ = instrumental analytical response (GC-MS) of a spiked sample; D_unspiked_ = instrumental analytical response (GC-MS) of a unspiked sample; C_spike_ = known concentration (μg/g) of the standard added to the sample.

R% has been applied to correct the result and to define the actual values of the concentration of each analyte (PAEs) in the samples, through the equation S2 (Table S1).

| **PAEs** | **% R *U. lactuca*** | **% R Sediments** |
| --- | --- | --- |
| **DMP** | 20,00 | 71 |
| **DEP** | 35,00 | 111 |
| **DBP** | 46,00 | 161 |
| **BBP** | 56,00 | 111 |
| **DEHP** | 84,00 | 82 |
| **DnOP** | 79,00 | 72 |

**Table S1**. Average recovery percentages of 6 phthalates from *U. lactuca* and sediment samples

In addition, the total phthalate concentration measured (total PAEs) was calculated for each sample analysed, expressed in (μg/g) according to equation S3.

[PAE] (mg/kg) = 100 * D_unspiked_ / R (eq.S2)

Total PAEs (mg/kg) = [DMP] + [DEP] + [DBP] + [BBP] + [DEHP] + [DnOP] (eq.S3)

| **PAEs** | **RT (min)** | **MRM** | **Dwell time (ms)** | **Collision energy (eV)** |
| --- | --- | --- | --- | --- |
| **DMP** | 2.4 | 163 , 133 | 30 | 10 |
| **DEP** | 2.7 | 149 , 65 | 30 | 30 |
| **DBP** | 3.6 | 149 , 65  223 , 149 | 30  30 | 30  10 |
| **BBP** | 4.8 | 149 , 65 | 30 | 30 |
| **DEHP** | 5.4 | 149 , 65  279 , 149 | 30  30 | 30  20 |
| **DnOP** | 6.3 | 149 , 65  279 , 149 | 30  30 | 30  20 |

**Instrumental parameters of GC/MS**

Flow: 1mL/min; oven temperature: 220°C to 310°C; MSD transfer line temperature: 270°C; source temperature: 250°C; electron energy -70eV; solvent delay 2.25min; pressure 28.2 psi, designed considering the fragments of each PAEs reported in Table S1.

**Table S2.** Retention time (RT), fragments set in multiple reaction monitoring (MRM), Dwell time and Collision energy parameters used for each phthalate (PAEs).

Regarding temperature ramp condition, initial oven temperature was programmed at 220°C (hold for 1 min), followed by a 40°C/min ramp to 310°C (hold for 5 min). Total run time was 8.25 min and the injection volume was 1 µL per sample. Quality checks were performed by analysing a 500 ng/mL (500 ppb) of a standard solution every four samples preceded and followed by a blank solvent injection.

The samples were injected in duplicate, with the sample injections intervalled with injections of blank solvent solution of ACN to avoid cross-contamination. In any case, the instrumental program provided for the washing of the syringe with n-hexane three times before the injection and once after it.

In some cases, traces of BBP, DEHP and DnOP were detected in blank analyses (not more than 1 ppb) and were accounted for by subtracting the integral value from the sample analysis chromatogram.

**Sampling campaign and pre-experiment operations**

The samples of *Ulva lactuca* consisted of small fragments leaves (3-5 cm), collected near the bay of Solanto (Palermo - Sicily - Mediterranean Sea) in an area of 20 x 10 meters (38 º 4' 37.88' N; 13 º 32' 33.83'' E) during the month of May 2021 together with sediments (gravel of particle size < 0.5 cm in diameter). Samples of algae and sediment were collected manually a few centimeters from the surface within 10 meters from the coastline, while seawater was taken at a depth of 5 meters 1 mile away from the coast. All samples were sealed in previously cleaned glass jars/bottles with three washing cycles (Acetonitrile-Hexane-Acetonitrile) to avoid contamination. Seaweeds samples were immediately taken to the laboratory and stored inside an aquarium (volume: 50 L) equipped with an oxygenator placed inside a thermostatic chamber, (16 °C). In order to recreate a cycle of natural light, a neon (45W; 15cm; 18’’; 4500K) was used, connected to a timer set with 12 hours of light and 12 hours of darkness.

In this way the samples were housed for a total of seven days before the start of the experiment. The sediments and seaweed samples were checked and washed with the same seawater used for the entire experiment to remove unwanted material (small branches, shells, fragments of other vegetal matrices, etc). In order to carry out an assessment of the initial pollution status, 20 *U. lactuca* fragments were collected and analysed, showing a contamination range for each phthalate from 0 to 1 ppm.

|  |  | Timeline | | | | | |
| --- | --- | --- | --- | --- | --- | --- | --- |
|  |  | First sampling (5th day) | | | | Second sampling (12th day) | Third sampling (12th day) |
|  |  | hυ irradiation | | | |  |  |
| # glass tube  (microcosms) | Concentration (mg/L) | 0 h | 0.5 h | 1.5 h | 8 h |  |  |
| 1 | 0 | ANALYSIS | ANALYSIS |  |  |  |  |
| 2 | 0 | ANALYSIS | ANALYSIS |  |  |  |  |
| 3 | 0 | ANALYSIS |  | ANALYSIS |  |  |  |
| 4 | 0 | ANALYSIS |  | ANALYSIS |  |  |  |
| 5 | 0 | ANALYSIS |  |  | ANALYSIS |  |  |
| 6 | 0 | ANALYSIS |  |  | ANALYSIS |  |  |
| 7 | 0 |  |  |  |  | ANALYSIS |  |
| 8 | 0 |  |  |  |  | ANALYSIS |  |
| 9 | 0 |  |  |  |  |  | ANALYSIS |
| 10 | 0 |  |  |  |  |  | ANALYSIS |
| 11 | 5 | ANALYSIS |  |  |  |  |  |
| 12 | 5 | ANALYSIS |  |  |  |  |  |
| 13 | 5 |  |  |  |  | ANALYSIS |  |
| 14 | 5 |  |  |  |  | ANALYSIS |  |
| 15 | 5 |  |  |  |  |  | ANALYSIS |
| 16 | 5 |  |  |  |  |  | ANALYSIS |
| 17 | 10 | ANALYSIS | ANALYSIS |  |  |  |  |
| 18 | 10 | ANALYSIS | ANALYSIS |  |  |  |  |
| 19 | 10 | ANALYSIS |  | ANALYSIS |  |  |  |
| 20 | 10 | ANALYSIS |  | ANALYSIS |  |  |  |
| 21 | 10 | ANALYSIS |  |  | ANALYSIS |  |  |
| 22 | 10 | ANALYSIS |  |  | ANALYSIS |  |  |
| 23 | 10 |  |  |  |  | ANALYSIS |  |
| 24 | 10 |  |  |  |  | ANALYSIS |  |
| 25 | 10 |  |  |  |  |  | ANALYSIS |
| 26 | 10 |  |  |  |  |  | ANALYSIS |
| 27 | 50 | ANALYSIS |  |  |  |  |  |
| 28 | 50 | ANALYSIS |  |  |  |  |  |
| 29 | 50 |  |  |  |  | ANALYSIS |  |
| 30 | 50 |  |  |  |  | ANALYSIS |  |
| 31 | 50 |  |  |  |  |  | ANALYSIS |
| 32 | 50 |  |  |  |  |  | ANALYSIS |
| 33 | 100 | ANALYSIS | ANALYSIS |  |  |  |  |
| 34 | 100 | ANALYSIS | ANALYSIS |  |  |  |  |
| 35 | 100 | ANALYSIS |  | ANALYSIS |  |  |  |
| 36 | 100 | ANALYSIS |  | ANALYSIS |  |  |  |
| 37 | 100 | ANALYSIS |  |  | ANALYSIS |  |  |
| 38 | 100 | ANALYSIS |  |  | ANALYSIS |  |  |
| 39 | 100 |  |  |  |  | ANALYSIS |  |
| 40 | 100 |  |  |  |  | ANALYSIS |  |
| 41 | 100 |  |  |  |  |  | ANALYSIS |
| 42 | 100 |  |  |  |  |  | ANALYSIS |

**Table S3**. Schematic description of the experimental setup

**Control experiment**


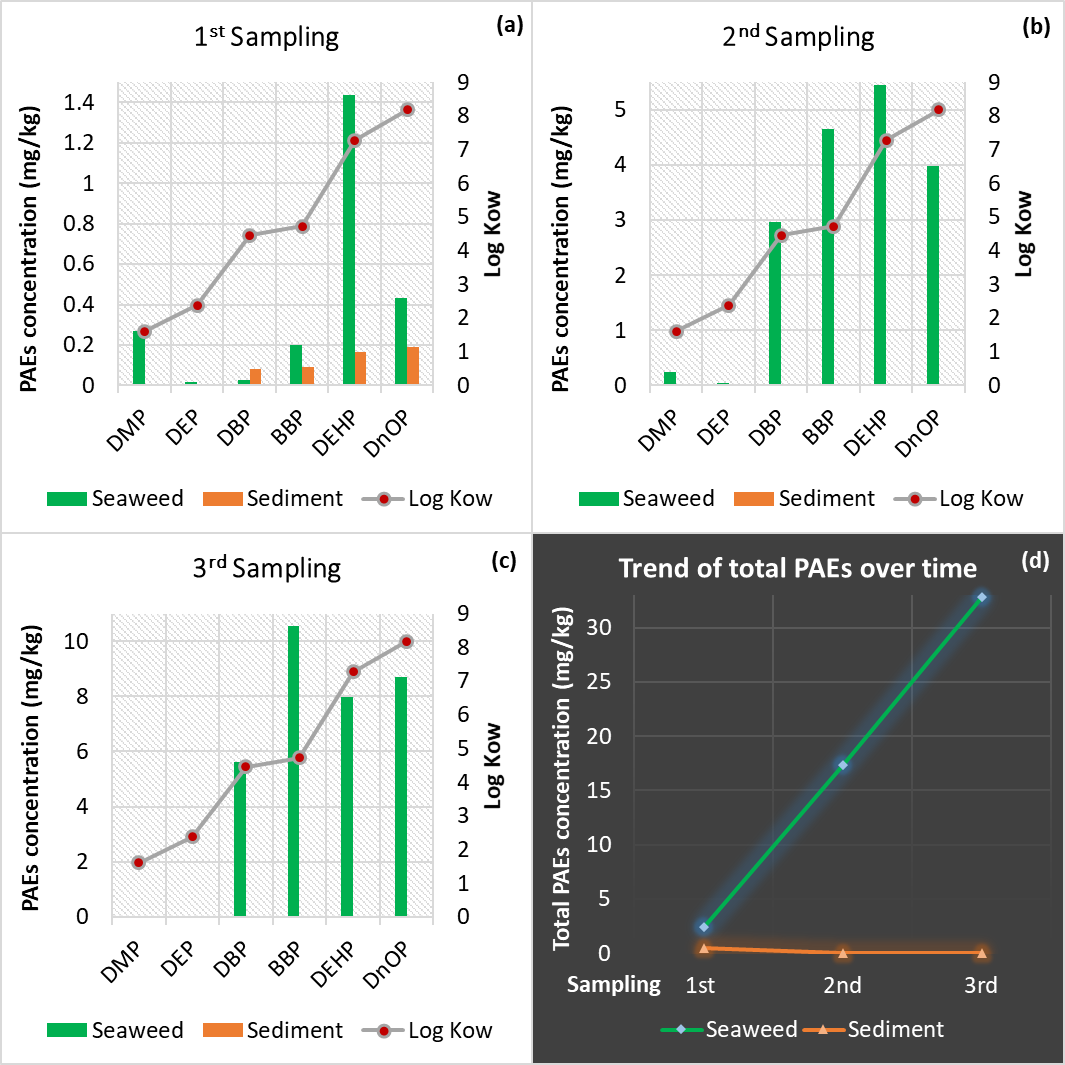


**Figure S1.** Control experiment (0 ppm): concentration values for each of the six phthalates (x-axis) (a,b,c) represented by the boxes relative to the scale of values on the left in the y-axis, compared to the respective octanol-water partition coefficient (log K_ow_) (represented by the red dots in the line relative to the values to the right of the Y axis). Concentration values of total sum of the six phthalates (total PAEs) (d) measured in *U. lactuca* samples and sediments analysed at first (a), second (b) and third (c) sampling and trend of total PAEs (d)

**Sampling**

**5 ppm experiment**


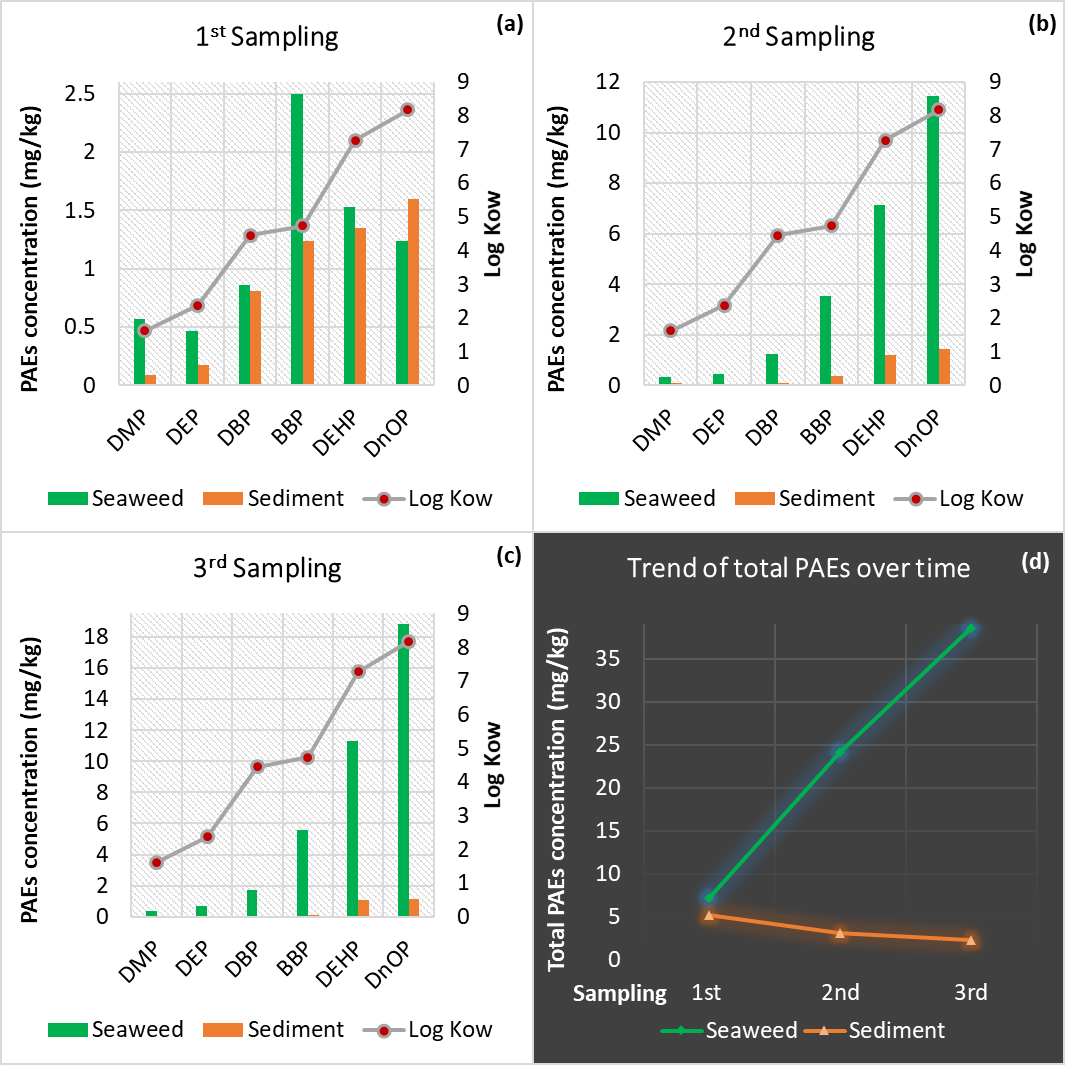


**Figure S2.** 5 ppm experiment: concentration values for each of the six phthalates (x-axis) (a,b,c) represented by the boxes relative to the scale of values on the left in the y-axis, compared to the respective octanol-water partition coefficient (log K_ow_) (represented by the red dots in the line relative to the values to the right of the Y axis). Concentration values of total sum of the six phthalates (total PAEs) (d) measured in *U. lactuca* samples and sediments analysed at first (a), second (b) and third (c) sampling and trend of total PAEs (d).

**Sampling**

**10 ppm experiment**


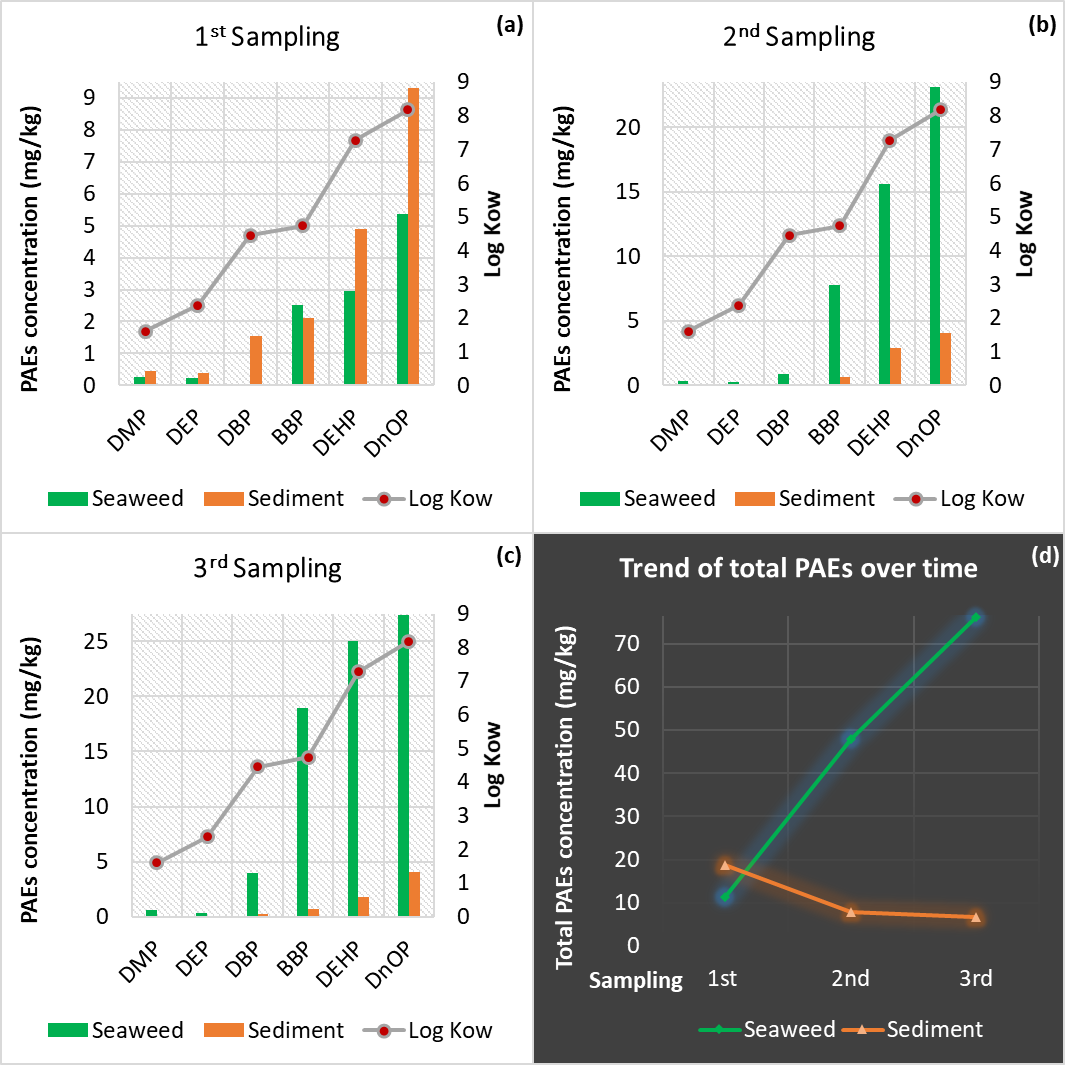


**Figure S3.** 10 ppm experiment: concentration values for each of the six phthalates (x-axis) (a,b,c) represented by the boxes relative to the scale of values on the left in the y-axis, compared to the respective octanol-water partition coefficient (log K_ow_) (represented by the red dots in the line relative to the values to the right of the Y axis). Concentration values of total sum of the six phthalates (total PAEs) (d) measured in *U. lactuca* samples and sediments analysed at first (a), second (b) and third (c) sampling and trend of total PAEs (d).

**Sampling**

**50 ppm experiment**


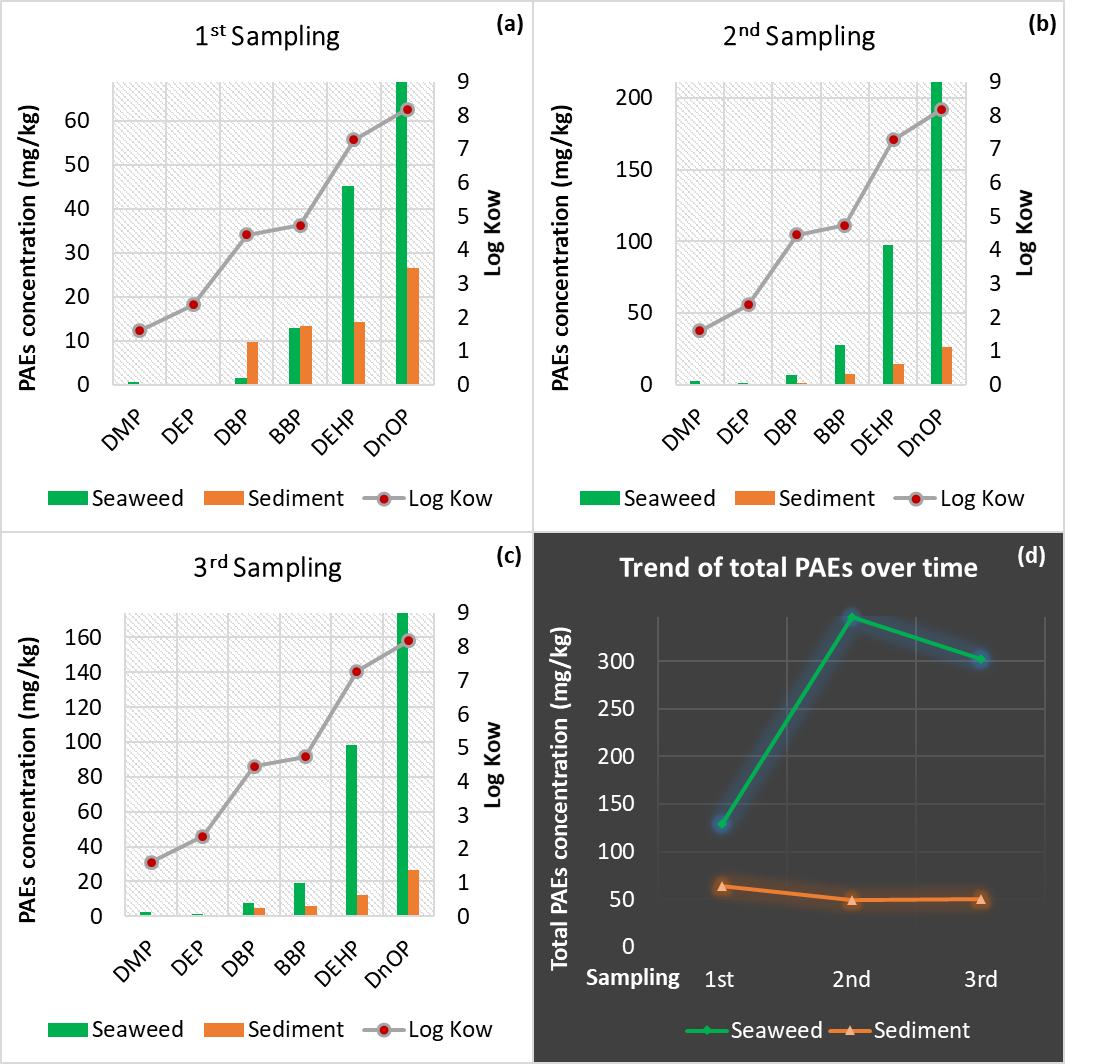


**Figure S4.** 50 ppm experiment: concentration values for each of the six phthalates (x-axis) (a,b,c) represented by the boxes relative to the scale of values on the left in the y-axis, compared to the respective octanol-water partition coefficient (log K_ow_) (represented by the red dots in the line relative to the values to the right of the Y axis). Concentration values of total sum of the six phthalates (total PAEs) (d) measured in *U. lactuca* samples and sediments analysed at first (a), second (b) and third (c) sampling and trend of total PAEs (d).

**Sampling**

**100 ppm experiment**

**
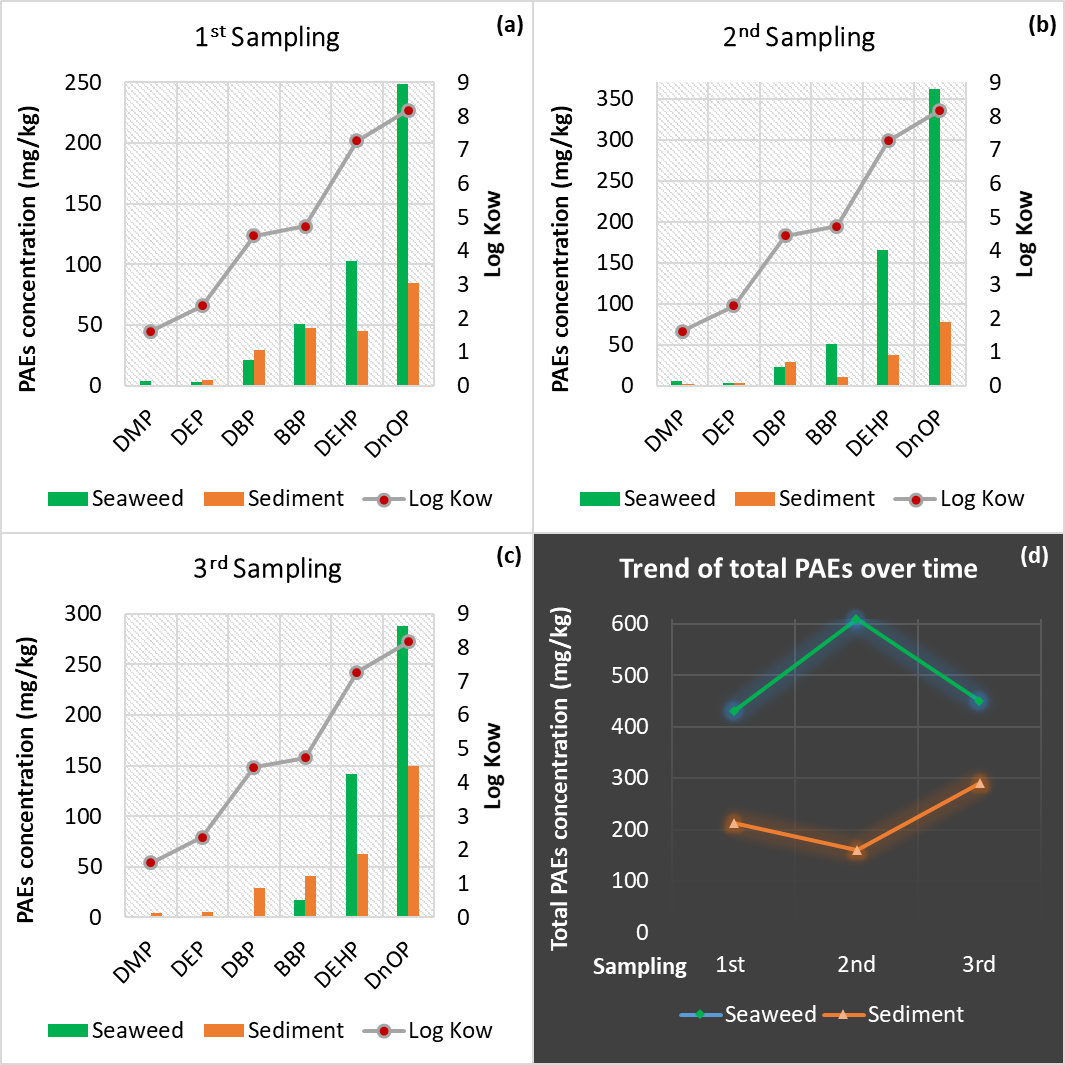
**

**Figure S5.** 100 ppm experiment: concentration values for each of the six phthalates (x-axis) (a,b,c) represented by the boxes relative to the scale of values on the left in the y-axis, compared to the respective octanol-water partition coefficient (log K_ow_) (represented by the red dots in the line relative to the values to the right of the Y axis). Concentration values of total sum of the six phthalates (total PAEs) (d) measured in *U. lactuca* samples and sediments analysed at first (a), second (b) and third (c) sampling and trend of total PAEs (d).

**Sampling**
